# Anatomical location of volar wrist ganglion in preoperative MRI is a risk factor for operation-related complications after arthroscopic ganglionectomy

**DOI:** 10.1186/s12891-025-08766-x

**Published:** 2025-07-01

**Authors:** Won-Taek Oh, Hyun-Kyo Kim, Do-Hyun Kim, Jae-Yong Cho, Il-Hyun Koh, Yun-Rak Choi

**Affiliations:** Department of Orthopedic Surgery, Yonsei University College of Medicine, 50-1, Yonsei-ro, Seodaemun-gu, Seoul, Seoul, 03722 Korea

**Keywords:** Volar wrist ganglion, Arthroscopic ganglionectomy, Postoperative complications, Risk factors

## Abstract

**Background:**

This study aimed to analyze risk factors of operation-related complications after arthroscopic ganglionectomy in patients with volar wrist ganglions, including patients’ demographics and ganglions’ anatomical characteristics in MRI. We hypothesized that volar wrist ganglions, either located distal to the bifurcation of the radial artery or superficially expanded, would associate with complications after arthroscopic ganglionectomy.

**Methods:**

This retrospective study included patients who had an arthroscopic ganglionectomy for volar wrist ganglion from Mar 2012 to Feb 2022 and followed up over one year. We reviewed medical records to gather patients’ demographics. The preoperative MRI was also examined to analyze the anatomical characteristics of the ganglion, involving axial location, superficial expansion, size, and presence of multiple lesions. The axial location was separated into two entities, whether located proximally or distally from the bifurcation of the radial artery. The superficial expansion was categorized into three depending on deep and superficial fascia penetration. For operation-related complications, we included the partial injury of the radial artery, median or dorsal branch of the radial nerve, and recurrence of ganglions after surgery.

**Results:**

Forty-five patients were enrolled in this study. The partial injury of the radial artery occurred in four patients(8.9%); two were ligated, and others were repaired intraoperatively. The recurrence has occurred in two patients(4.4%). On univariate logistic regression analysis, these complications were associated with the anatomical location of the ganglion when it was distal to the bifurcation of the radial artery and concurrently penetrated up to the superficial fascia layer(*p* = 0.035). The others were unrelated to complications, including revision surgery, multiple lesions, size, and anatomical locations unless it was concurrent.

**Conclusions:**

The operation-related complications after arthroscopic volar wrist ganglionectomy are associated with its anatomical location when distal to the bifurcation of the radial artery and concurrently penetrated up to the superficial fascia layer.

**Trial registration:**

Retrospectively registered.

## Introduction

Volar wrist ganglions occupy approximately 30% of wrist ganglions, the second most common to the dorsal wrist ganglions [1, 2]. Surgical excision should be considered when patients have pain or cosmetic concerns due to volar wrist ganglions, though it is usually unnecessary for asymptomatic ones [2–4]. Arthroscopic excision has been recognized as a proper surgical option with several advantages, such as the minimal invasiveness and the efficient resection of communicating fluid-filled stalk between ganglion cysts and the radiocarpal joint, although current data are limited to demonstrate the superiority of arthroscopic excision to open excision [1, 2, 5].

Despite the increasing popularity of arthroscopic excision, it could not be free from recurrence and other operation-related complications. The recurrence rate after arthroscopic excision has averaged 6% in previous studies, although it is lower than the average 21% of open excision [1, 6]. Other complications have also been reported after arthroscopic volar wrist ganglionectomy, including injuries of the radial artery, the palmar cutaneous branch of the median nerve, the cutaneous branch of the ulnar nerve, and the superficial radial nerve [2, 7, 8]. Because each volar wrist ganglion has different anatomical characteristics, this diversity in ganglion location, size, and multiple lesions could be associated with postoperative complications. However, there is a lack of studies analyzing these variables as risk factors for postoperative complications after volar wrist ganglionectomy.

This study aimed to analyze risk factors for operation-related complications, including patients’ demographics and ganglions’ anatomical characteristics in MRI. We hypothesized that volar wrist ganglions, either located distal to the bifurcation of the radial artery or superficially expanded, would be associated with complications after arthroscopic ganglionectomy.

## Materials and methods

This retrospective study included patients who had an arthroscopic ganglionectomy for volar wrist ganglion from Mar 2012 to Feb 2022 and followed up over one year. Patients with the following criteria were excluded: (1) unavailable preoperative MRI, (2) previous surgery on the ipsilateral wrist except for open excision of volar wrist ganglion, (3) combined disease around the ipsilateral wrist, and (4) inadequate follow-up. The same hand surgeon (Y.C) performed arthroscopic volar wrist ganglionectomy during the study period.

### Medical reviews and MRI analysis

One observer (W.O.) retrospectively reviewed all medical records to gather patients’ demographics, including age, gender, duration, and past surgical history. For operation-related complications, the following were retrieved according to previous studies: the partial injury of the radial artery, flexor tendon, median nerve, or dorsal branch of the radial nerve and recurrence of ganglions after surgery [3, 6, 8]. To report clinical outcomes after volar wrist ganglionectomy, we also assessed preoperative and postoperative clinical outcomes, including visual analog scale (VAS) pain score, grip strength, range of motion (ROM), Mayo wrist score (MWS), and Disabilities of Arm, Shoulder, and Hand (DASH). The VAS score for wrist pain was defined as the severity of pain experienced by patients and ranged from 0 (no pain) to 10 (worst possible pain). Active flexion-extension and radial-ulnar deviation arcs of the wrist were measured using a handheld goniometer. Grip strength was measured using a Jamar hydraulic dynamometer (Asimov Engineering, Los Angeles, CA, USA). The MWS is a commonly used wrist rating system, and its total score ranges between 0 and 100 points, with higher scores indicating better results. This system consists of 4 categories: pain (25 points), active flexion-extension arc as a percentage of the opposite side (25 points), grip strength as a percentage of the opposite side (25 points), and the ability to return to regular employment or activities (25 points). The DASH score is based on self-reported answers to a questionnaire designed by Davis et al. [9], which contains 30 items: 21 questions that assess difficulties with specific tasks, 5 questions that evaluate symptoms, and 4 questions that assess social function, work function, sleep, and confidence. The DASH score ranges between 0 and 100, with higher scores representing greater upper extremity disability.

**Fig. 1:**
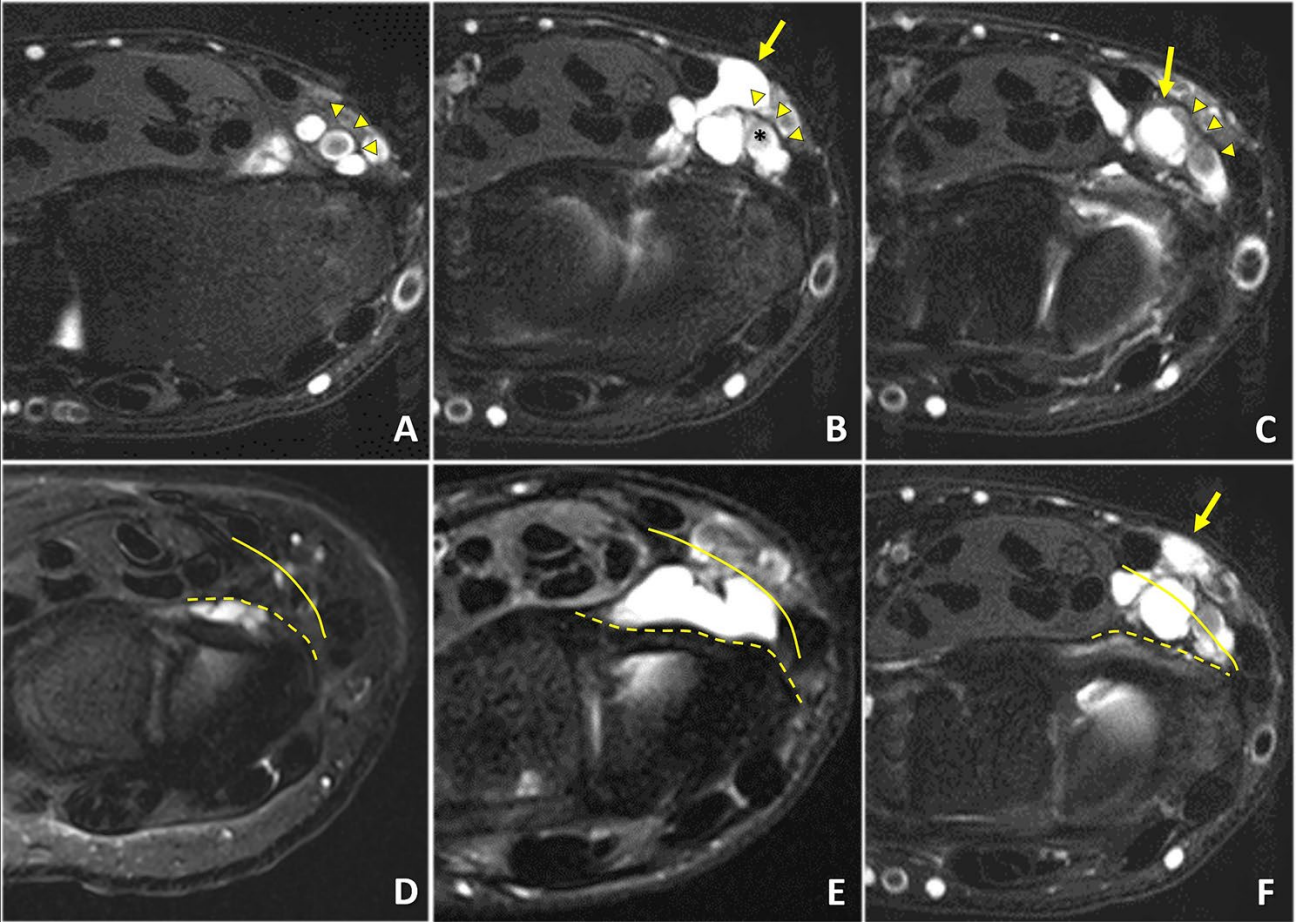
Anatomical characteristics of the volar wrist ganglion in preoperative MRI. (**A-C**) The axial location of the ganglion based on the bifurcation (*) of the radial artery (arrowheads). This patient’s volar wrist ganglion was distal to the bifurcation (arrow). (**D-F**) The superficial expansion of the ganglion depending on the penetration of the deep fascia (dashed line) and superficial fascia (solid line). This patient’s ganglion expanded up to the superficial fascia (arrow)

Two surgeons (W.O. and J.C.), who were uninvolved in the patient treatment, examined preoperative MRIs to analyze the anatomical characteristics of the ganglion, involving axial location, superficial expansion, size, and multiple lesions. The axial location was separated into two entities, whether located proximally or distally from the bifurcation of the radial artery. The bifurcation point of the radial artery was defined when a clear round appearance of the radial artery in axial view was lost and changed into oval or asymmetric. We classified them as “proximal” when the center of the ganglion was proximal to the bifurcation and “distal” when distally located (Fig. 1A-C). The superficial expansion was categorized into three depending on deep and superficial fascia penetration (Fig. 1D-F). The first layer was within the deep fascia, the same layer with volar capsuloligamentous structures and the pronator quadratus. The second was between the superficial and deep fascia, the same layer with the flexor digitorum profundus and the flexor policis longus. The last was above the superficial fascia, located with the flexor carpi radialis and the radial artery. To confirm our hypothesis, we also classified the “combined” group when the ganglion was located distal to the bifurcation and above the superficial layer simultaneously.

### Surgical technique

**Fig. 2:**
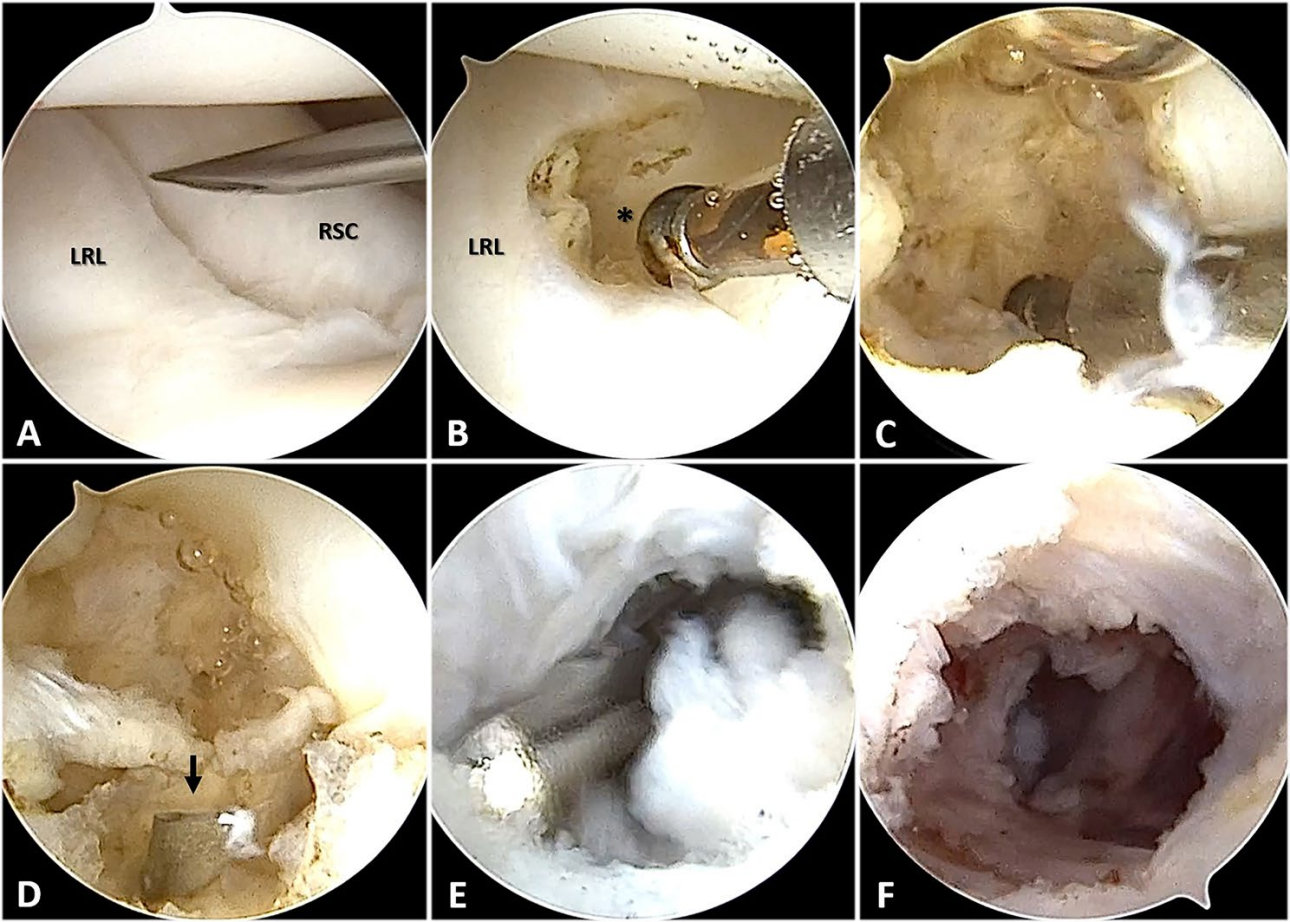
Arthroscopic excision of the volar wrist ganglion. (**A**, **B**) The entry point for ganglionectomy was established between LRL and RSC using the 3–4 portal as a viewing portal and the 1–2 portal as a working portal. (**C**) Arthroscopic ganglionectomy was conducted with a motorized shaver (2.0-mm) and radiofrequency ablation. (**D**, **E**) An additional volar portal (arrow, guide needle) was created, and debridement of the ganglion sac was performed as much as possible. (**F**) Arthroscopic excision of the volar wrist ganglion was accomplished. LRL, Long radiolunate ligament; RSC, Radioscaphocapitate ligament

Under general anesthesia with the patient supine, each patient’s arm was prepared and draped on a hand table and exsanguinated using an Esmarch bandage and tourniquet. The patient’s arm was suspended in an ARC wrist tower (Acumed, Hillsboro, OR) using 5 to 8 kg of traction after placing the index, middle, and ring fingers in finger traps. A 3–4 portal was established as a viewing portal, and a 1–2 portal as a working portal. An initial entry point to remove ganglions was created between the radioscaphocapitate(RSC) ligament and the long radiolunate(LRL) ligament, from which most volar wrist ganglions originated (Fig. 2A, B). A radiofrequency ablator and motorized 2.0-mm shaver were employed to enlarge the entry (3 to 5 mm size) and debride the juxta-articular capsuloligamentous structures (Fig. 2C). After that, an additional volar portal, which could reach the debrided area, was created to remove the sac of the ganglion further (Fig. 2D-F). A vessel bleeding or a damaged tendon was thoroughly examined with the pneumatic tourniquet deflated. After the operation, a compressive dressing and a wrist brace were used. Wrist motion and progressive strengthening exercises were initiated 2 weeks after the surgery.

### Statistical analysis

All statistical computations relied on standard software (R freeware v4.2.0; R Foundation for Statistical Computing, www.r-project.org). The paired t-test or Wilcoxon rank-sum test was used to compare pre and postoperative VAS pain score, grip strength, active range of wrist motion, MWS, and DASH score. Simple logistic regression was used for risk factor analysis. Statistical significance was set at a p-value less than *0.05*.

## Results

During the study period, there were 59 patients who had undergone an arthroscopic ganglionectomy for volar wrist ganglion. Fourteen patients were excluded from analysis after examining medical records and imaging studies (unavailable preoperative MRI, *n* = 3; previous surgery on the ipsilateral wrist except for open excision of volar wrist ganglion, *n* = 2; combined disease around the ipsilateral wrist, *n* = 3; inadequate follow-up, *n* = 6). Forty-five patients were finally enrolled in this study. The mean follow-up duration was 17.8 ± 10.5 (12 to 48) months. Among clinical outcomes, VAS pain, flexion-extension arc, MWS, and DASH score were improved postoperatively (Table 1). The partial injury of the radial artery occurred in four patients (8.9%); two were ligated, and others were repaired intraoperatively. The recurrence has occurred in two patients (4.4%). On univariate logistic regression analysis, these complications were associated with the anatomical location of the ganglion when it was distal to the bifurcation of the radial artery and concurrently penetrated up to the superficial fascia layer (p = *0.035*). The others were unrelated to complications, including revision surgery, multilocation, size, and anatomical locations unless it was concurrent (Table 2).

**Table 1:** Preoperative and postoperative clinical outcomes after arthroscopic volar ganglionectomy

Variables	Preoperative	Postoperative	*P* value*
VAS pain	5.0 ± 2.6	1.3 ± 1.5	< 0.001†
Grip strength (%)	88.1 ± 20.8	92.9 ± 21.1	0.126
Flexion-extension arc (°)	130.1 ± 11.4	134.7 ± 14.6	0.003†
Supination-pronation arc (°)	169.0 ± 8.4	169.6 ± 6.8	0.395
Radial-ulnar deviation arc (°)	54.3 ± 7.2	55.8 ± 8.7	0.185
MWS	77.6 ± 13.7	88.3 ± 10.2	< 0.001†
DASH score	22.9 ± 13.7	8.1 ± 7.4	< 0.001†

Continuous values are mean ± standard deviation. Percentage values are the percentage of the contralateral side. VAS = Visual Analogue Scale; MWS = Mayo Wrist Score; DASH = Disabilities of Arm, Shoulder, and Hand. *P values are calculated using the Paired t-test or the Wilcoxon rank sum test for continuous variables. †*P* < 0.05

**Table 2:** Simple logistic regression for Operation-related complications after arthroscopic volar ganglionectomy

Variables	B	SE	95% CI	OR or MD	*P* value*
Demographic					
Age (yr)	0.06	0.03	-0.01–0.12	10.10	0.088
Gender, female	-1.97	1.14	-4.21–0.27	0.14	0.084
Revision	-15.80	NA	NA	NA	0.994
MRI					
Length (mm)	0.10	0.25	-0.05–0.55	2.08	0.102
Axial location†	1.35	1.14	-0.89–3.59	3.87	0.237
Superficial expansion ‡	18.31	NA	NA	NA	0.994
Combined§	2.42	1.15	0.17–4.67	11.25	0.035¶
Number of ganglions, >3	1.46	1.14	-0.78–3.69	4.28	0.202

B, estimate; SE, standard error; 95% CI, 95% confidence interval; OR, odds ratio; MD, mean difference. †Distal to the bifurcation of the radial artery. ‡Superficially expanded above the superficial fascia. §Distal to the bifurcation of the radial artery and expanded above the superficial fascia simultaneously. *P values are calculated using Simple logistic regression. ¶*P* < 0.05

## Discussion

Arthroscopic excision of volar wrist ganglion is a favorable surgical treatment with the advantage of minimal invasiveness and cosmetic benefit. However, this technique also accompanies operation-related complications, and there have been many articles reporting nerve, artery, and tendon injuries and recurrence of ganglion after the operation [1, 2, 5]. In this study, we retrospectively analyzed the volar wrist ganglion patients who had an arthroscopic excision, including preoperative MRI, and found that operation-related complications were associated with the anatomical location of the ganglion. The ganglion, superficial to radial artery and distal to radial artery bifurcation, was susceptible to intraoperative radial artery injury or postoperative recurrence. This is the first study to investigate the risk factors for operation-related complications after arthroscopic volar wrist ganglion excision.

In previous studies, the recurrence rate after arthroscopic volar wrist ganglionectomy ranged from 0 to 13% in patients with a mean follow-up of over 2 years [2, 4, 10–12]. This is similar to the recurrence rate of our study, which was 4.4% (2 out of 45 patients). Although there was a paucity of studies analyzing risk factors for postoperative recurrence, two explanations were provided. Konigsberg et al. argued that the surgeon’s experience might affect recurrence rates, showing that the recurrence rates decreased over time in their retrospective cohort studies, which included 3 fellowship-trained surgeons performing arthroscopic ganglionectomy for 3 years [13]. However, this argument is inappropriate to our study because the recurred cases in ours were dispersed throughout the study period. Several authors have noted that capsulectomy, which creates a capsular defect at the radioscaphocapitate-long radiolunate interval, is insufficient, especially when the mucinous ganglion material is uncertain intraoperatively and extended debridement outside the joint capsule involving the ganglion sac, may be required [2, 5, 12]. However, they recommended that surgeons should avoid extending too far volar due to the risk of injury to the radial artery or the sensory branch of the median nerve.

Previous clinical articles have reported that operation-related complications after arthroscopic volar wrist ganglionectomy averaged 7.1% (0 − 8.5%) [2, 4, 10–12, 14]. Most complications were related to the radial artery (4 out of 122 cases) or peripheral sensory nerve (6 out of 122 cases). In our retrospective study, there were four cases of radial artery rupture intraoperatively, including two cases that required open repair, although no nerve injuries were reported. According to previous literature [2, 5], radial artery injury during arthroscopic ganglionectomy appears to be associated with the proximity of the ganglion to the radial artery, which is consistent with our finding of the ganglion’s anatomical location on the preoperative MRI. Our analysis showed that ganglions crossing the radial artery and located distal to the bifurcation were susceptible to injury. We suspected that the radial artery distal to the bifurcation might have a thinner wall and more complex anatomical variations than the proximal portion, making it vulnerable to extensive debridement of the ganglion sac.

Our study has several limitations. First, it is a retrospective study, and there could be a patient who had postoperative complications or recurrence but lost to follow-up. This could result in selection bias because we had excluded those patients. Second, it included a small number of patients, which could increase the uncertainty of our risk factor analysis. Third, this study analyzed risk factors for intraoperative complications and postoperative recurrence, focusing solely on patient factors. However, surgeon-related factors could also affect the outcome, including the surgeon’s experience, surgical instruments, and even the size of the capsuloligamentous opening being ablated during the procedure. Although the same senior surgeon performed all surgeries using identical procedures during our study period, this could have been a confounding factor.

## Conclusions

The operation-related complications after arthroscopic volar wrist ganglionectomy are associated with its anatomical location: distal to the bifurcation of the radial artery and concurrently penetrated up to the superficial fascia layer.

## Data Availability

No datasets were generated or analysed during the current study.
